# Exaggerated renal fibrosis in P2X4 receptor-deficient mice following unilateral ureteric obstruction

**DOI:** 10.1093/ndt/gfu019

**Published:** 2014-02-25

**Authors:** Min Jeong Kim, Clare M. Turner, Reiko Hewitt, Jennifer Smith, Gurjeet Bhangal, Charles D. Pusey, Robert J. Unwin, Frederick W.K. Tam

**Affiliations:** 1 Imperial College Renal and Transplant Centre, Hammersmith Hospital, Imperial College London, London,UK; 2 Clinic for Transplantations immunology and Nephrology, University Hospital Basel, Basel, Switzerland; 3 Department of Biomedicine, Molecular Nephrology, University Hospital Basel, Basel, Switzerland; 4 UCL Centre for Nephrology, University College London, London, UK

**Keywords:** connective tissue growth factor, P2X4 receptor, renal fibrosis, TGF-β, unilateral ureteric obstruction

## Abstract

**Background:**

The ATP-sensitive P2X7 receptor (P2X7R) has been shown to contribute to renal injury in nephrotoxic nephritis, a rodent model of acute glomerulonephritis, and in unilateral ureteric obstruction (UUO), a rodent model of chronic interstitial inflammation and fibrosis. Renal tubular cells, endothelial cells and macrophages also express the closely related P2X4 receptor (P2X4R), which is chromosomally co-located with P2X7R and has 40% homology; it is also pro-inflammatory and has been shown to interact with P2X7R to modulate its pro-apoptotic and pro-inflammatory effects. Therefore, we chose to explore the function of P2X4R in the UUO model of renal injury using knockout mice. We hypothesized that UUO-induced tubulointerstitial damage and fibrosis would also be attenuated in P2X4R^−/−^ mice.

**Method:**

P2X4R^−/−^ and wild-type (WT) mice were subjected to either UUO or sham operation. Kidney samples taken on Days 7 and 14 were evaluated for renal inflammation and fibrosis, and expression of pro-fibrotic factors.

**Results:**

To our surprise, the obstructed kidney in P2X4R^−/−^ mice showed more severe renal injury, more collagen deposition (picrosirius red staining, increase of 53%; P < 0.05) and more type I collagen staining (increase of 107%; P < 0.01), as well as increased mRNA for TGF-β (increase of 102%, P < 0.0005) and CTGF (increase of 157%; P < 0.05) by Day 14, compared with the UUO WT mice.

**Conclusion:**

These findings showed that lack of P2X4R expression leads to increased renal fibrosis, and increased expression of TGF-β and CTGF in the UUO model.

## INTRODUCTION

Extracellular purines and pyrimidines are important signalling molecules that mediate diverse biological effects via cell surface receptors known as purine receptors [1]. There are two main types of purinergic receptors, P1 and P2: the ligand for P1 is adenosine and for P2 ATP (adenosine 5′-triphosphate), ADP (adenosine 5′-diphosphate), UDP (uridine 5′-diphosphate) and UTP (uridine 5′-triphosphate). P2 receptors are divided into two subclasses, P2X and P2Y: P2X are ligand-gated ion channels and P2Y are G protein-coupled receptors. Among the seven mammalian P2X receptors, P2X7 (P2X7R) has been shown to have pro-apoptotic and pro-inflammatory functions in many tissues, including the kidney [2], as well as a potentially proliferative and pro-fibrotic function, demonstrated in the nephrotoxic nephritis (NTN) [3] and unilateral ureteral obstruction (UUO) [4] models, respectively. Turner *et al.* [2] reported up-regulation of P2X7R in human lupus-related GN and in rodent models of GN. Taylor *et al.* [3] found that in the NTN model of glomerulonephritis, P2X7R gene deficiency was reno-protective when compared with wild-type (WT) controls; in addition, the selective P2X7R antagonist A-438079 prevented development of NTN in rats. Moreover, Gonçalves *et al*. [4] showed in the UUO model that P2X7R deficient mice had significantly attenuated tubulointerstitial injury.

Compared with P2X7R, the role of the chromosomally adjacent and structurally related P2X4 receptor (P2X4R) in kidney injury is unknown [5]. P2X4R has a wider tissue distribution than P2X7R, including in blood vessels, lung, kidney, and, like P2X7R, immune cells [6]. P2X4R has been detected at the mRNA level, and as expressed protein in a variety of renal cell types, including glomerular mesangial [7] and epithelial cells [8] and most tubular cells, especially proximal tubular cells [7, 9], but also collecting duct cells [10, 11], although it is worth noting that P2 receptor subtypes and their distribution can vary by species [12]. However, P2X4R function along the nephron is still unclear, but at least two recent studies have suggested a role in affecting distal nephron sodium transport [13–15]. So far its function has been studied mainly in the central and peripheral nervous systems, and in the vasculature. P2X4R is involved in synaptic transmission in neurons and has excitatory effects when bound to extracellular ATP. Increased P2X4R expression in microglia has been observed after spinal cord injury [16] and brain ischaemia [17]. In human vascular endothelial cells, P2X4R is involved in ATP-induced Ca^2+^ influx [18] and flow-mediated vasodilatation through nitric oxide release, affecting blood pressure and vascular remodelling [19].

Published data have suggested that P2X4R and P2X7R may exist as heterotrimers in certain tissues, including bone marrow-derived macrophages [20], although interactions between homotrimeric P2X7R and P2X4R seem more likely [21]. Brone *et al.* [22] found evidence for P2X4 and P2X7 ion channel currents in recruited peritoneal macrophages, and that both receptors are expressed, and can functionally interact, in murine macrophages [23]. Recently, Kawano *et al.* [23] have shown that P2X7R-mediated inflammation is regulated by co-expression with P2X4R through facilitation of IL-1β release from a mouse macrophage cell line. Moreover, Gu *et al.* [24] have provided evidence in the eye for an interaction between P2X7R and P2X4R that determines the phenotypic function of the macrophage and its clearance of apoptotic cells, which could in turn affect ATP release from necrotic cells following tissue damage. While the role of P2X7R in renal pathology has been the main focus of recent interest, a potential role for P2X4R has remained relatively unexplored; however, findings to date suggest a close functional relationship between P2X4R and P2X7R, and therefore a possible role for P2X4R in renal injury [11].

In the present study, we have examined the role of P2X4R in the UUO model of chronic inflammation and fibrosis using P2X4R knockout (KO) mice. We chose this model because a previous study in UUO in P2X7R KO mice had shown a reduction in renal inflammation and fibrosis, and we had expected to find similar protection in P2X4R KO mice; however, surprisingly, we observed an increase in renal fibrosis.

## MATERIALS AND METHODS

### Animals

P2X4R-deficient mice were gifts from GlaxoSmithKline and they have been described in detail elsewhere [25]. Age- and sex-matched WT mice of the same genetic background (C57BL/6) were used as controls. These mice were obtained from Charles River UK animal suppliers (Kent, UK). The growth rates of these animals were indistinguishable from those of WT animals, and mutant mice reproduced normally. All mice were male and aged 10–12 weeks. Animals had free access to standard laboratory diet and water. Mice were kept in a pathogen-free environment, and experiments were performed according to institutional and UK Home Office guidelines.

### Experimental design

The animals underwent either sham-operation or UUO operation. Under sterile conditions, animals were anaesthetized with a mixture of isoflurane and oxygen. Two ties were knotted around the mid-portion of the left ureter, using a thin non-absorbable suture (5/0, Mersilk) and the ureter was sectioned between the ligatures. The abdomen was closed with running sutures and the skin was closed with interrupted sutures. Sham-operated animals underwent identical surgical procedures, except that the left ureter was manipulated without ligation and sectioning. The animals were killed at 7 days or 14 days after UUO. Each group consisted of six mice.

### Tissue preparation

Samples of kidney were either unfixed or fixed in periodate-lysine-paraformaldehyde (PLP) and snap-frozen in isopentane precooled in liquid nitrogen, or fixed in neutral-buffered formalin and then embedded in paraffin for sectioning. Tissues fixed in neutral-buffered formalin were used for periodic-acid Schiff (PAS) staining, Sirius-red staining (SR) and immunohistochemistry for alpha-smooth muscle actin and collagen. Snap-frozen tissues fixed in PLP were used for CD68 staining for macrophage detection.

### Immunohistochemistry

To stain for CD68, endogenous peroxidase was blocked by 3% hydrogen peroxide for 10 min and then tissues were incubated with a rat anti-mouse CD68 antibody (Serotec Ltd, Oxford, UK, MCA 1957) for 1 h. For the detection, Polink-2 HRP plus rat DAB detection system (Dako, D46–15) was used and the counterstain was done with haematoxylin. For detection of α-smooth muscle actin (α-SMA) and collagen I, standard immunohistochemical techniques were used. In brief, tissue sections were rehydrated through xylene and graded alcohols, boiled with 0.01 M sodium citrate buffer for 15 min, and then sections were blocked for endogenous peroxidase (Dako peroxidase block, 10 min, room temperature). Sections were blocked in 10% Marvel milk for 30 min. The primary antibodies were rabbit anti-α-SMA (1:500, ab 5694; Abcam, Cambridge, UK), rabbit anti-collagen I (1:500, ab 34710; Abcam, Cambridge, UK) and rabbit anti-P2X4R antibody (1:2000, APR-002; Alomone labs, Jerusalem, Israel). They were diluted in 1% goat serum and incubated for up to 6 h at room temperature. For detection, Dako envision kit with labelled polymer conjugated to goat anti-rabbit IgG (K 4011; Dako, Ely, Cambridgeshire, UK) was used.

### Histology

In PAS-stained sections, tubulointerstitial injury was assessed semi-quantitatively in a blinded fashion (modified from Ophascharoensuk *et al.*) [26]. For each animal, 20 consecutive high-power fields were scored for the presence of inflammatory cells within the interstitium by the presence of tubular dilatation, atrophy, and cast formation, and by the presence of tubular basement membrane thickening and interstitial widening (no damage = score 0; damage in <5% of HPF = score 1; damage in 5–<25% of HPF = score 2; damage in 25–50% of HPF = score 3; damage in >50% of HPF = score 4). The mean tubulointerstitial damage score was calculated and used for statistical analysis.

For the analysis for SR, CD68, α-SMA and collagen I, five consecutive fields of cortex and five fields of medulla were captured under ×200 magnification using a Phototonic Science Color Coolview camera (Photonic Sciences, Robertsbridge, UK), and analysed using Image Pro 7 software (Media Cybernetis, Silver Spring, MD, USA). Images were converted to grey-scale 256-bit images for the analysis for SR. A single observer performed blinded morphological measurements.

### Real-time quantitative PCR

Total RNA from kidney was isolated using TRIzol reagents (Invitrogen, Paisley, UK). Two micrograms of RNA was reverse-transcribed with the First Strand cDNA Synthesis Kit for RT–qPCR (AMV) (Roche Applied Science, Burgess Hill, UK). Real-time quantitative PCR (RT–qPCR) was performed on the Mastercycler® ep realplex (Eppendorf, Histon, UK) using the SYBR green master-mix (Thermo Scientific, Loughborough, UK). GAPDH or 18s rRNA served as the internal control. The sequences of PCR primers used in this study are listed in Table 1. The expression of mRNA was analysed by a relative quantification method, the 2-ΔΔCt method.

**Table 1.** Primers used in real-time RT–PCR amplification

Gene	Primer nucleotide sequences
MCP-1	Forward 5′-CCTCTGGGCCTGCTGTTCA-3′
	Reverse 5′-CCAGCCTACTCATTGGGATCA-3′
TGF-β	Forward 5′-GCAACATGTGGAACTCTACCAGAA-3′
	Reverse 5′-GACGTCAAAAGACAGCCACTCA-3′
CTGF	Forward 5′-CAAAGCAGCTGCAAATACCA-3′
	Reverse 5′-GGCCAAATGTGTCTTCCAGT-3′
Fibronectin	Forward 5′-ACACGGTTTCCCATTACGCCAT-3′
	Reverse 5′-AATGACCACTGCCAAAGCCCAA-3′
GAPDH	Forward 5′-GCATGGCCTTCCGTGTTC-3′
	Reverse 5′-GATGTCATCATACTTGGCAGGTTT-3′
P2X4R	Forward 5′-CCCTTTGCCTGCCCAGATAT-3′
	Reverse 5′-CCGTACGCCTTGGTGAGTGT-3′
18s rRNA	Forward 5′-ACCGCGGTTCTATTTTGTTG-3′
	Reverse 5′-CCCTCTTAATCATGGCCTCA-3′

MCP-1, monocyte chemoattractant protein-1; TGF-β, transforming growth factor-β; CTGF, connective tissue growth factor; GAPDH, glyceraldehyde 3-phosphate dehydrogenase; 18s rRNA, 18s ribosomal ribonucleic acid.

### Statistical analysis

Data are given as mean ± S.D. Differences between groups were analysed by Mann–Whitney *U*-test. All probabilities were two tailed. P-values <0.05 were considered significant. Statistical analyses were performed using Prism 5.0 (GraphPad, Software, La Jolla, CA, USA).

## RESULTS

### Expression of P2X4R

**FIGURE 1:**
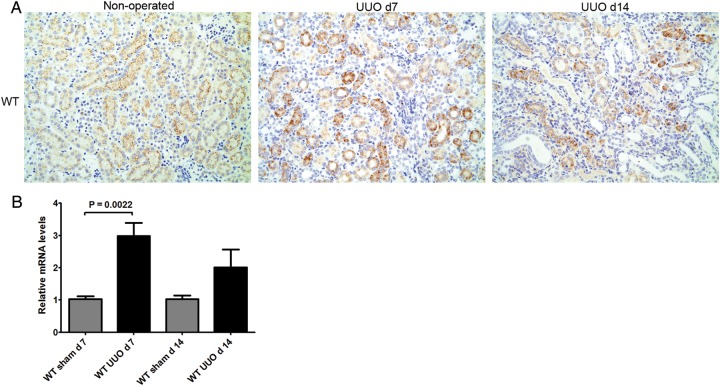
Expression of P2X4R following UUO: (**A**) Immunohistochemistry for P2X4R in WT mice following UUO: There was a linear positive staining on the luminal surface of tubular epithelial cells in non-operated kidneys, whereas the positive staining was internalized after 7 and 14 days in UUO-operated kidneys. Original magnification ×70. (**B**) Increased expression of P2X4R mRNA following UUO detected by RT–qPCR.

Expression of P2X4R protein and mRNA in renal tissue of WT mice was studied by immunohistochemistry and RT–qPCR, respectively. P2X4R protein was detected in kidney of sham-operated WT mice (Figure 1A), showing linear positive staining for P2X4R on the luminal surface of tubular epithelial cells. Diffuse P2X4R expression was detected on tubular epithelial cells at 7 and 14 days after induction of UUO in the WT mice (Figure 1A). A significant increase in P2X4R mRNA was detected 7 days after induction of UUO (P = 0.0022) compared with sham-operated mice (Figure 1B).

### Tubulointerstitial injury

After 7 and 14 days of ureteral obstruction, UUO-operated kidneys in all WT mice showed the typical features of obstructive nephropathy, with tubular dilatation and cast formation, and widespread tubulointerstitial damage, inflammation and fibrosis.

**FIGURE 2:**
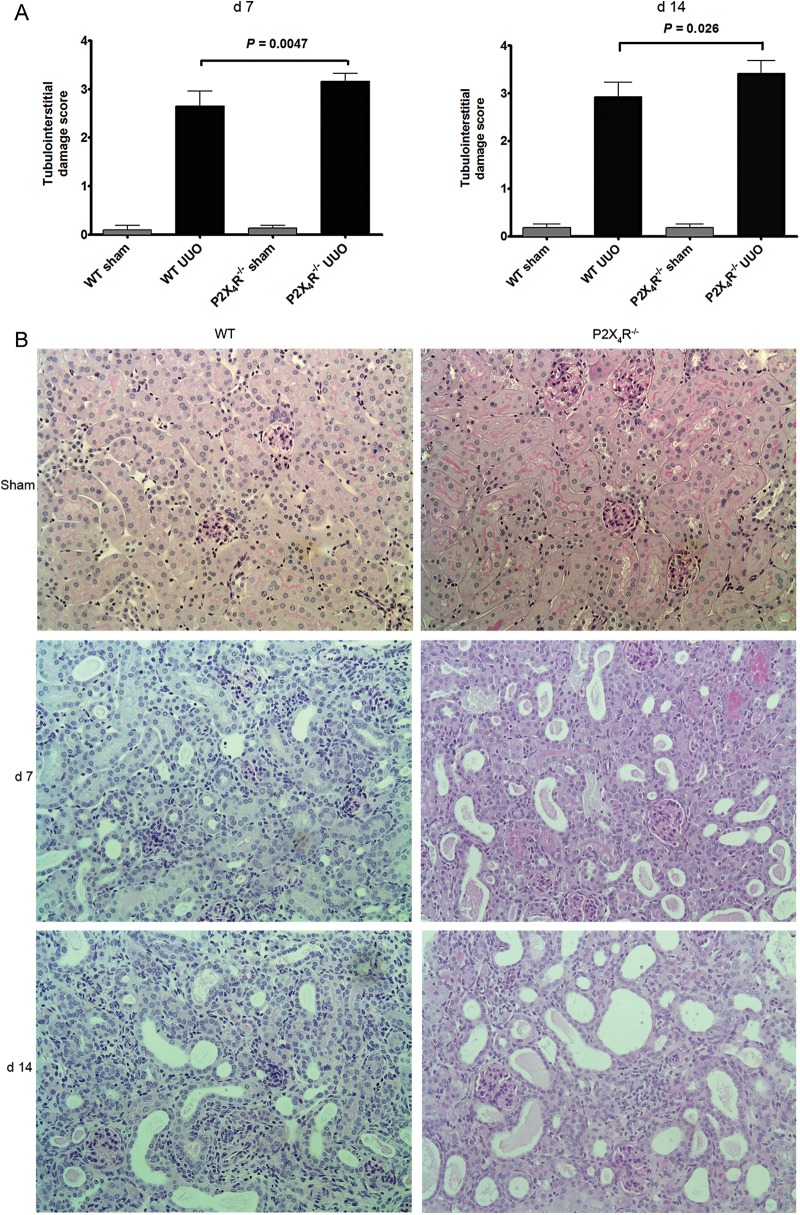
Tubulointerstitial injury following UUO: (**A**) Scoring of tubulointerstitial damage is significantly higher in P2X4R−/− mice, in comparison with WT mice after 7 and 14 days of UUO. (**B**) Histological findings of UUO kidney sections after 7 and 14 days of UUO stained by periodic-acid Schiff. Original magnification ×80.

Tubulointerstitial injury was assessed and scored on PAS-stained tissues according to the severity of injury. There was a significant increase in tubulointerstitial injury in P2X4R^−/−^ UUO mice compared with WT UUO mice on Days 7 (P = 0.0047) and 14 (P = 0.026) (Figure 2A). No difference in tubulointerstitial injury was detected between sham-operated kidneys from P2X4R^−/−^ and WT mice on Days 7 and 14. Figure 2B shows the typical histological findings of obstructed kidneys in each UUO group.

Next we assessed the potential influences of P2X4R on the development of tubulointerstitial fibrosis following UUO.

### Tubulointerstitial fibrosis

#### Extracellular matrix

**FIGURE 3:**
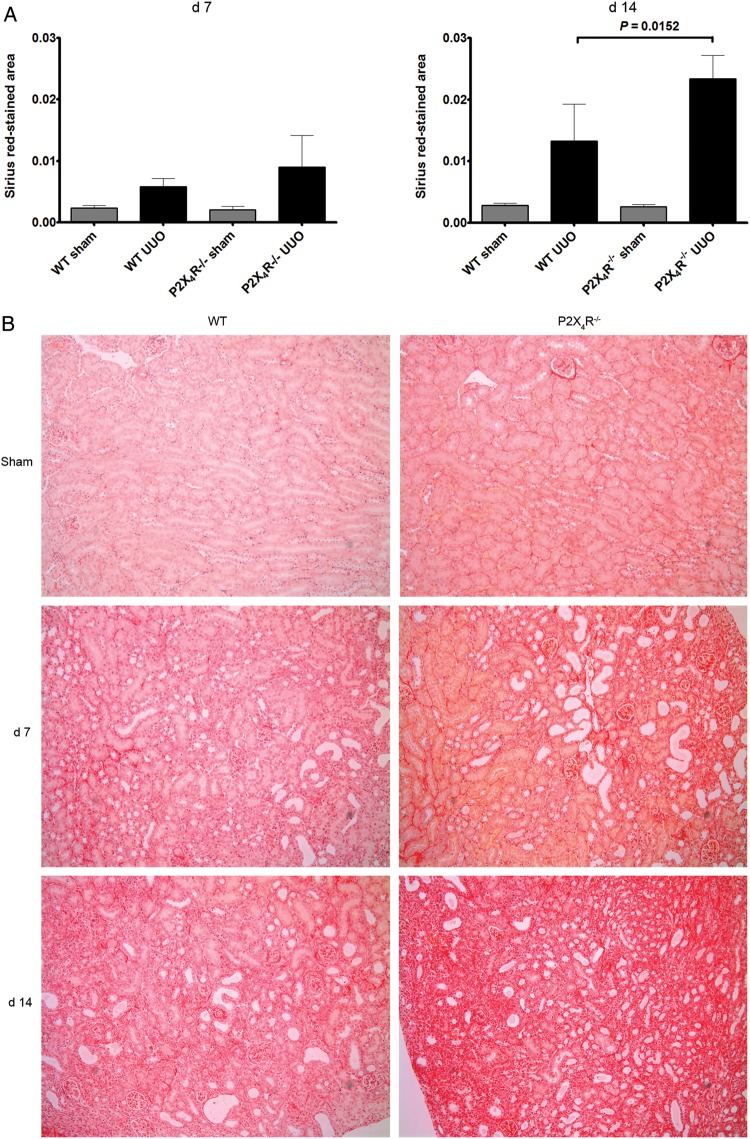
Effect of deficiency of P2X4R in the synthesis of collagen after UUO: (**A**) Sirius-red (SR) staining in P2X4R−/− was significantly greater compared with WT by Day 14 as quantified by image analysis. (**B**) SR staining of UUO kidney sections on Day 7 and 14 in different groups. Original magnification ×70.

**FIGURE 4:**
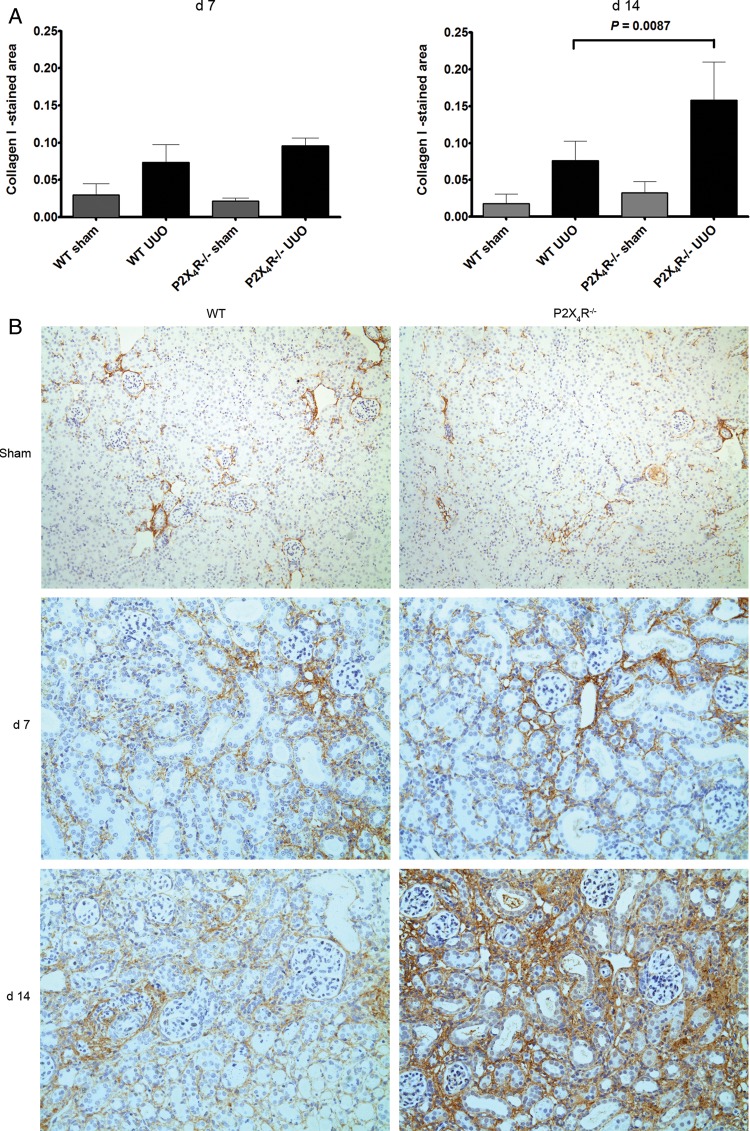
Effect of deficiency of P2X4R in the synthesis of collagen I after UUO: (**A**) Immunostaining for collagen I was significantly greater in P2X4R−/− compared with WT by Day 14. (**B**) Immunostaining of UUO kidney sections for collagen I on Day 7 and 14. Original magnification ×80.

Since UUO-operated kidneys typically show increased interstitial expression of extracellular matrix, we examined the expression of collagen and fibronectin in kidney tissue. We examined expression of collagen by SR staining. Collagen fibrils were identified using this staining method at Days 7 and 14. There was no significant difference in SR staining between sham-operated P2X4R^−/−^ and WT kidneys. There was a progressive increase in SR staining in UUO from Day 7 to Day 14 in WT and P2X4R^−/−^ mice; however, SR staining on Day 14 in UUO-operated kidneys was significantly increased in P2X4R^−/−^ mice compared with WT UUO (increase of 53%; P = 0.0152) (Figure 3A and B). To verify the SR staining results, we examined the expression of collagen I by immunohistochemistry (Figure 4A and B). The pattern of collagen I staining was consistent with SR staining. Day 14 UUO-operated kidneys from P2X4R^−/−^ mice expressed more collagen I (increase of 107%; P = 0.0087) when compared with WT UUO mice.

**FIGURE 5:**
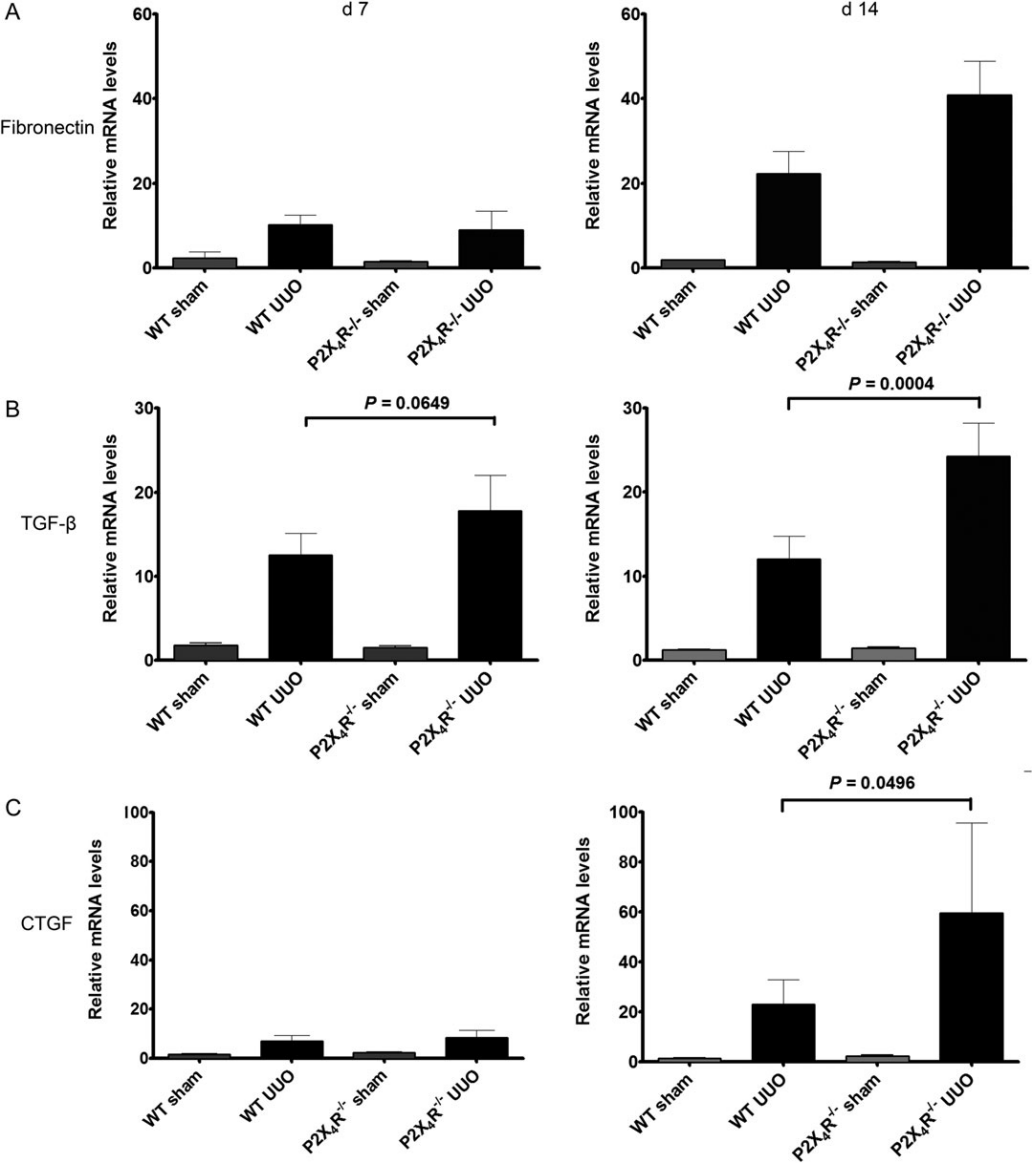
Effect of deficiency of P2X4R in the expression of fibronectin, TGF-β and CTGF in UUO: (**A**) The increase in expression of fibronectin mRNA in P2X4R−/− was not statistically significant in comparison with WT by Day 14. (**B**) The expression of TGF-β mRNA was significantly higher in P2X4R−/− compared with WT on Day 14. (**C**) By Day 14 the expression of CTGF mRNA was significantly higher in P2X4R−/− compared with WT.

To investigate the renal expression of fibronectin, renal mRNA expression was examined by real-time RT–qPCR on Days 7 and 14. There was no significant difference in fibronectin mRNA expression between sham-operated kidneys of WT and P2X4R^−/−^ animals (Figure 5A). By Day 7, WT and P2X4R^−/−^ UUO mice showed higher expression of fibronectin mRNA than sham-operated mice; however, there was no significant difference between WT and P2X4R^−/−^ UUO mice. By Day 14, there was 84% higher expression of fibronectin mRNA in P2X4R^−/−^ UUO mice compared with WT UUO mice, although this did not reach statistical significance.

#### Pro-fibrotic growth factors

Since transforming growth factor-β (TGF-β) and connective tissue growth factor (CTGF) play a significant role in the progression of renal fibrosis in both clinical and experimental renal disease, we examined the renal expression of TGF-β and CTGF mRNA by real-time RT–PCR on Days 7 and 14. The renal expression of TGF-β mRNA in UUO-operated kidneys was higher in P2X4R^−/−^ mice compared with WT mice by Day 7 (increase of 21%; P = 0.065) and by Day 14 (increase of 102%; P = 0.0004) (Figure 5B). No significant differences in TGF-β mRNA expression were seen in sham-operated kidneys from WT or P2X4R^−/−^ mice. The pattern of expression of CTGF mRNA was similar to that of TGF-β. The UUO-operated kidneys expressed significantly higher CTGF mRNA levels than the sham-operated kidneys. By Day 14, CTGF mRNA was up-regulated in UUO-operated kidneys of P2X4R^−/−^ mice compared with WT UUO mice (increase of 157%; P = 0.0496). There was no significant difference in CTGF mRNA expression between sham-operated kidneys from WT or P2X4R^−/−^ (Figure 5C). Higher expression of both TGF-β and CTGF mRNA in P2X4R^−/−^ mice following UUO suggests a pro-fibrotic effect of P2X4R deficiency.

#### Alpha-smooth muscle actin

**FIGURE 6:**
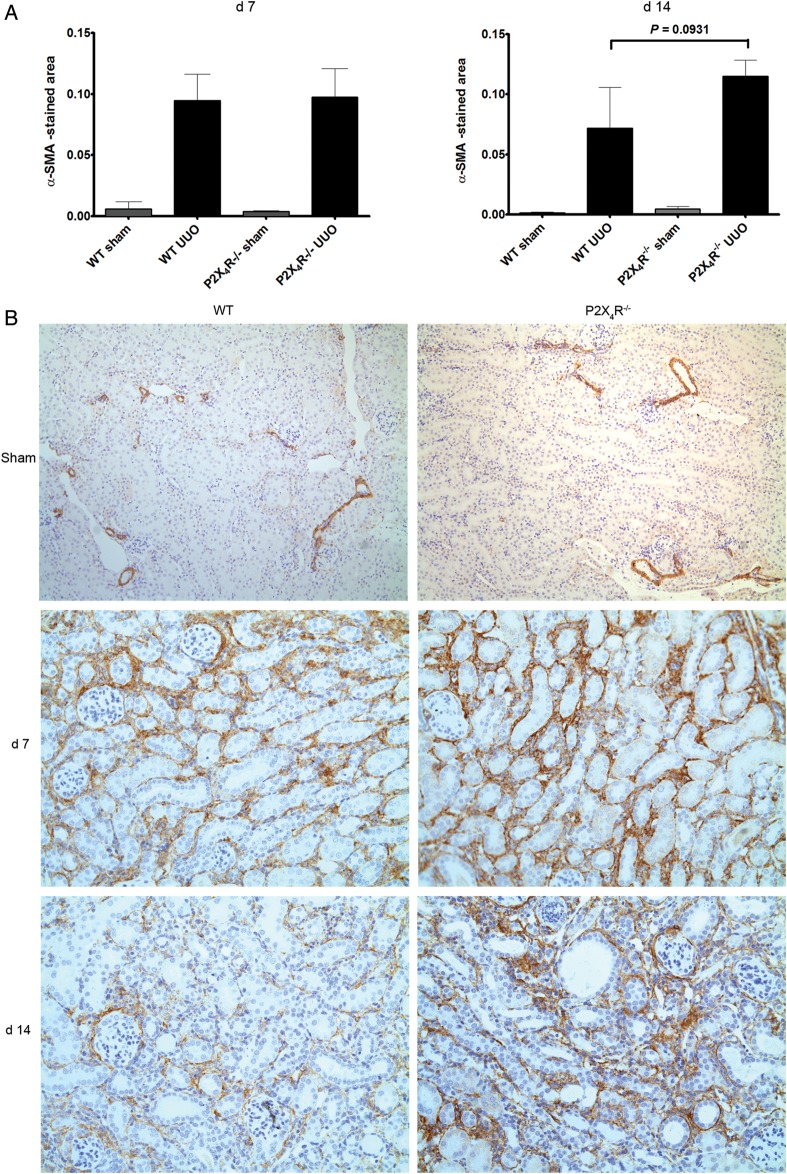
Effect of deficiency of P2X4R in the expression of α-SMA after UUO: (**A**) Immunostaining for α-SMA in P2X4R−/− compared with WT on Day 7. The increase in α-SMA staining in P2X4R−/− only showed a trend. (**B**) Immunostaining of UUO kidney sections for α-SMA on Day 7 and 14 in UUO kidneys. Original magnification ×80.

Since myofibroblasts may be involved in renal fibrosis, we performed immunohistochemistry for α-SMA (Figure 6A and B). UUO-operated kidneys expressed significantly higher α-SMA than sham-operated kidneys on Days 7 and 14 in both groups of animals. α-SMA-positive staining in UUO-operated kidneys was 60% higher in P2X4R^−/−^ mice compared with WT mice on Day 14, although the differences were not statistically significant (P = 0.09).

The results of the analyses for renal collagen were consistent with the expression of the pro-fibrotic growth factors TGF-β and CTGF. This finding indicates that deficiency of P2X4R has a significant effect on the promotion of tubulointerstitial fibrosis following UUO.

#### Renal MCP-1

**FIGURE 7:**
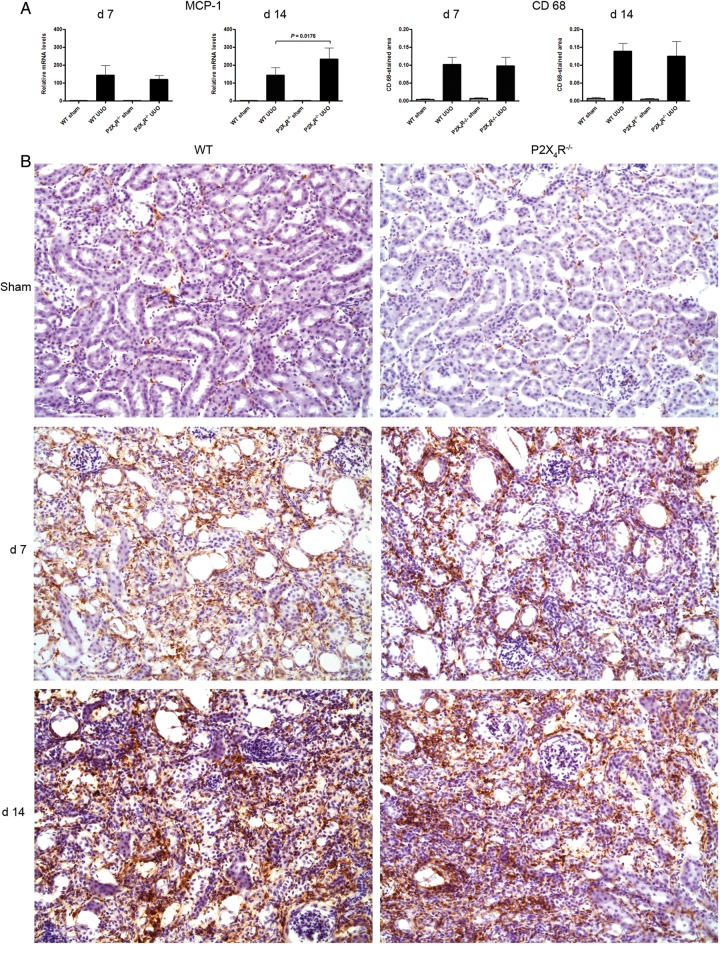
Effect of deficiency of P2X4R on renal MCP-1 and macrophages after UUO: (**A**) There was no significant differences in MCP-1 mRNA expression on Day 7 (WT or P2X4R−/−). By day MCP-1 14 mRNA expression was significantly higher in P2X4R−/− compared with WT. (**B**) Immunostaining for CD68 shows no significant differences between different groups on both Day 7 and Day 14.

We examined whether deficiency of P2X4R affects renal expression of CCL2 [monocyte chemoattractant protein-1 (MCP-1)]—a chemokine for macrophages—mRNA by RT–qPCR. As shown in Figure 7A, MCP-1 mRNA was highly expressed in UUO-operated kidneys compared with sham-operated kidneys on Days 7 and 14. On Day 7, UUO-operated kidneys from P2X4R^−/−^ and WT mice showed no significant difference in MCP-1 mRNA expression, but by Day 14, UUO-operated kidneys from P2X4R^−/−^ mice expressed 62% higher MCP-1 mRNA levels compared with WT mice (P = 0.0176). In the sham-operated kidneys, there was no significant difference in MCP-1 mRNA expression between P2X4R^−/−^ and WT kidneys on Day 7 or 14.

#### Renal macrophages

Macrophages have a role in inflammation and fibrosis, and express P2X4R, as well as P2X7R. Immunohistochemistry of renal tissue for macrophages using anti-CD68 antibody on kidney tissue from Days 7 and 14 after sham or UUO showed a significant increase in CD68-positive staining following UUO on Days 7 and 14 compared with sham-operated kidneys, but no significant difference between WT and P2X4R^−/−^ UUO on Day 7 or 14 (Figure 7).

## DISCUSSION

Inflammation and fibrosis underlie almost all forms of progressive renal disease, but what determines the balance and shift between inflammation and fibrosis is still poorly understood [27]. Thus, the focus of current research into renal pathophysiology has centred on these two key processes, and in trying to identify the underlying factors that may control or modify them.

Although P2X7R has been detected in renal tissue, including tubular cells, its expression is normally at a very low level [28], and any role for this receptor in the kidney seems more likely to depend on its expression by resident or infiltrating immune cells, particularly the macrophage [3]. Indeed, P2X7R deficiency has been shown to attenuate renal injury in rodent models of acute inflammatory glomerulonephritis [3], as well as in the UUO model [4], in DOCA-salt hypertension [29] and in a model of lupus nephritis [30]. In contrast, the function of P2X4R in the kidney is still unclear, but may include an effect on sodium transport [13–15]. As already mentioned, these receptors are structurally related and are often expressed in the same cell; several recent reports have suggested physical and functional interactions between these receptors when expressed by immune cells, which may affect the inflammatory response [9, 11, 31, 32]. It is perhaps also worth noting that in contrast to P2X7R, which has a low affinity for extracellular ATP and requires a high concentration for its activation, P2X4R is much more sensitive to ATP stimulation and can also form a non-lethal pore that is permeable to large organic molecules, but unlike P2X7R it can rapidly desensitize ATP [33]. However, what these biophysical properties may lead to in terms of biological function is still unclear.

Superficially at least, these receptors might be expected to have similar or overlapping functions; for example, both P2X7R and P2X4R have been shown to cause NLRP3 inflammasome activation [30, 34, 35]. Therefore, in the present study, we investigated the role of P2X4R in the development of renal inflammation and fibrosis following UUO, because of their frequent co-localization in the same cell, especially immune cells, as well as their structural, and possible functional, relationship [27, 36, 37]. The effect of a new generation of P2X4R antagonists has been reported from *in vitro* studies [38], but it is not known whether they are suitable for *in vivo* studies [39].

Thus, using P2X4R^−/−^ mice, we hypothesized that UUO-induced tubulointerstitial damage and fibrosis should be attenuated, as had been reported previously in P2X7R^−/−^ mice. However, we were surprised to find more tubulointerstitial damage and fibrosis in P2X4R KO mice: renal fibrosis increased significantly in P2X4R^−/−^ by Day 14 compared with WT mice, suggesting a pro-fibrotic effect in the absence of P2X4R. This observation is supported by the increased renal expression of TGFβ1 and CTGF mRNA, and of type I collagen deposition following UUO in P2X4R KO mice compared with WT mice. However, the number of infiltrating macrophages, which were similar on both Day 7 and Day 14, cannot explain the differences in fibrosis.

Immunohistochemistry for P2X4R showed linear positive staining on the luminal surface of tubular epithelial cells in non-operated kidneys, whereas staining for this receptor was more diffuse, including cytoplasmic, in UUO-operated kidneys. P2X4R has been detected both at the plasma membrane and in the cytoplasm of neurons, microglial cells and macrophages [21]. Stimulation of primary microglia cells with endotoxin for 3–6 h increased surface expression of P2X4R, probably by increasing trafficking to the membrane, as can MCP-1 stimulation [40], a chemokine that is also induced by P2X7R activation [3]. Exposure of neurons to extracellular ATP increases the internalization of P2X4R rapidly [41]. In the kidney, the trafficking of P2X4R from the cell surface to the cytoplasm may reflect a ligand-induced change during the tubulointerstitial responses to UUO, although how this might moderate the inflammatory response is unclear.

Cross-talk between renal epithelial cells has been demonstrated *in vitro* for the P2X7R mediating interstitial fibroblast cell death following tubular cell injury and release of ATP [42], but how P2X4R might be involved or modulate this effect has not been considered. The mesangial cell has some macrophage-like properties [43] and the macrophage itself may contribute to renal fibrosis. We did find significantly higher expression of MCP-1 mRNA in P2X4R^−/−^ mice by Day 14, but the number of infiltrating macrophages did not differ significantly between KO and WT mice, although their function may have altered.

Finally, a P2X4R-dependent vascular response might have affected renal blood flow, including the medullary circulation [44], which in turn could have altered renal tissue oxygenation, leading to more fibrosis in the UUO model. The P2X4R KO mouse is known to have a higher blood pressure and impaired flow-mediated vasodilatation from reduced endothelial-dependent nitric oxide release [19]. But again, we can only speculate as to what role this might play in UUO, although the presence of hypertension itself may predispose to more renal injury.

In conclusion, we report the unexpected finding that P2X4R deficiency increases renal fibrosis in the UUO model, which is in contrast to P2X7R. The accentuated fibrotic changes following UUO observed in P2X4R^−/−^ mice require further investigation, but serve to highlight a potential role for this receptor in renal pathology, and its relationship to P2X7R with which it may partner in regulating cell function when co-expressed.

## FUNDING

This project was supported by Wellcome Trust Project Grant (WT087435MA). M.J.K. was supported by Novartis Foundation for a research fellowship. F.W.K.T. was supported by Diamond Fund from Imperial College Healthcare NHS Trust.

## CONFLICT OF INTEREST STATEMENT

CDP has received research funding from Cyclacel, UCB Celltech and Glaxo Smith Kline. RJU is an external consultant to AstraZeneca Translational Medicine, Molndal, Sweden. FWKT has received research project grants from Roche Palo Alto, AstraZeneca Limited and Baxter Biosciences. The results presented in this paper have not been published previously in whole or part, except in abstract format.
